# Upgrading Biogas from Small Agricultural Sources into Biomethane by Membrane Separation

**DOI:** 10.3390/membranes11120938

**Published:** 2021-11-27

**Authors:** Aleksandra Janusz-Cygan, Jolanta Jaschik, Marek Tańczyk

**Affiliations:** Institute of Chemical Engineering, Polish Academy of Sciences, Ul. Bałtycka 5, 44-100 Gliwice, Poland; jjaschik@iich.gliwice.pl (J.J.); mtanczyk@iich.gliwice.pl (M.T.)

**Keywords:** biogas, biomethane, polysulfone and polyimide membranes, multicomponent membrane separation, mathematical modelling

## Abstract

The agriculture sector in Poland could provide 7.8 billion m^3^ of biogas per year, but this potential would be from dispersed plants of a low capacity. In the current study, a membrane process was investigated for the upgrading biogas to biomethane that conforms to the requirements for grid gas in Poland. It was assumed that such a process is based on membranes made from modified polysulfone or polyimide, available in the market in Air Products PRISM PA1020 and UBE UMS-A5 modules, respectively. The case study has served an agricultural biogas plant in southern Poland, which provides the stream of 5 m^3^ (STP) h^−1^ of biogas with a composition of CH_4_ (52 vol.%), CO_2_ (46.3 vol.%), N_2_ (1.6 vol.%) and O_2_ (0.1 vol.%), after a pretreatment. It was theoretically shown that this is possible to obtain the biomethane stream of at least 96 vol.% of CH_4_ purity, with the concentration of the other biogas components below their respective thresholds, as required in Poland for gas fuel “E”, with methane recovery of up to 87.5% and 71.6% for polyimide and polysulfone membranes, respectively. The energetic efficiency of the separation process is comparable for both membrane materials, as expressed by power excess index, which reaches up to 51.3 kW_th_ kW_el_^−1^ (polyimide) and 40.7 kW_th_ kW_el_^−1^ (polysulfone). In turn, the membrane productivity was significantly higher in the case of the polyimide membrane (up to 38.3 kW_th_ m^−2^) than those based on the polysulfone one (up to 3.13 kW_th_ m^−2^).

## 1. Introduction

The model of a circular economy and the European Union’s policy on biomass energy use are parts of the overall research and technological development strategy in Europe. Intensive research and development work is focused on new methods of processing biomass into components for motor fuels and chemicals [1,2,3,4,5,6]. Biogas, which is a by-product of the biological decomposition of organic substances under anaerobic conditions, belongs to the important renewable energy sources (RES) [1,7,8]. Apart from landfills and sewage treatment plants, it may come from biogas plants processing the agricultural or food industry waste. In Poland itself, the potential of the agri–food sector in terms of biogas generation is estimated at over 7.8 billion m^3^ per year [9]. Energy from biogas is particularly useful in the combined production of electricity and heat. Additionally, indeed, most of the biogas produced in Europe is burned in cogeneration units. However, due to the fact that biogas plants are located at a considerable distance from built-up areas, the cogenerated heat cannot be utilized at a satisfactory level. An alternative is to upgrade biogas to biomethane as an energy carrier. This can be generally carried out by absorption, adsorption, cryogenic or membrane separation [7,10,11,12,13,14]. Membrane processes compete with other separation methods by compact module setups, easy scaling-up of the continuous process, low energy consumption (due to the lack of phase transitions) and no need for additional sorbents [15,16,17,18,19,20,21]. As limitations, both the necessity to compress the gas to pressures of 1–2 MPa [7,8,12,15,22,23,24,25] and the initial preparation of biogas [7,10,11,12,14,22,23] are mentioned.

Membrane processes in the context of biogas enrichment have been investigated intensively for a decade and have already found practical application in large-scale installations with a capacity of several hundred m^3^ h^−1^ of biogas, operating at a pressure higher than 1 MPa [7,15,22,26]. Such processes consist of several stages, including raw gas preparation (H_2_S and solid particles removal, dehydration), compression and membrane separation [22]. In Poland the membrane biogas upgrading from various sources, including agricultural waste, has been studied, among others, by Chmielewski et al. [20]. However, an installation which produces biomethane from biogas is still to be implemented in the country, while in 2019 there were 725 such units in Europe [27], including 173 membrane setups [28]. Among the main reasons for the slow development of the biogas and/or biomethane market in Poland are the lower than expected level of support for investors, low social awareness of the benefits of using biogas and the lack of clear legal and administrative conditions. Moreover, in Poland, small and dispersed farms prevail, where a biogas plant, if exists, has a small capacity of a few m^3^ h^−1^ of biogas. Therefore, an implementation of a biomethane upgrading technology is not just a matter of downgrading existing solutions. With such a small scale, the energetic efficacy and overall profitability of the investment may be harder to achieve.

The main gaseous components of biogas from agricultural waste are methane (53–85 vol.%), carbon dioxide (14–48 vol.%), nitrogen (0.5–7.5 vol.%) and oxygen (<1 vol.%) [11,12,19,20,23,25,29,30]. In turn, two main impurities of the raw agricultural biogas are water vapour (1–10 vol.%) and H_2_S (10–30,000 ppm). If biogas is to be injected into the natural gas transmission network, it should be standardized. Requirements concerning the grid gas vary significantly for different countries [14], therefore a prospective biogas upgrading technology has to be adapted to them. According to Polish regulations [31], gas fuel “E” should have the heat of combustion ≥ 10.56 kWh m^−3^, the total amount of combustible components ≥96 vol.%, less than 4 vol.% of nitrogen, 3 vol.% of CO_2_, 0.2 vol.% of oxygen and 5 ppm of H_2_S. In order to meet these requirements, it is possible to perform the separation of all six components listed above in a process which uses commercial cellulose acetate, polyimide, polysulfone or polycarbonate membranes [11,19,20,26]. However, due to the high risk of formation of corrosive compounds, it is still necessary to remove or at least significantly reduce the concentration of water vapor and H_2_S before the essential separation process.

The main goal of this study was to assess the possibility of reaching a biomethane quality which conforms to the requirements for grid gas fuel, while upgrading biogas from a small agricultural source in a membrane process. As a case study, an agricultural biogas plant in southern Poland provides a stream of 5 m^3^ (STP) h^−1^ of biogas with a composition of CH_4_ (52 vol.%), CO_2_ (46.3 vol.%), N_2_ (1.6 vol.%) and O_2_ (0.1 vol.%) after a pretreatment. In the membrane process investigated in this paper, two membranes—that is, polyimide and polysulfone—were considered, which are used in commercial UBE UMS-A5 and Air Products PRISM PA1020 modules, respectively. The performance of these modules were preliminary assessed in the experimental separation of CH_4_/CO_2_ mixtures. Then, the enrichment of methane in the four-component agricultural biogas stream was investigated theoretically, especially in terms of required membrane area and the excess of thermal power that is carried with the biomethane stream, over the electric power necessary to carry out the essential separation process. The model developed and validated for the capture of CO_2_ from flue gases in a membrane and hybrid process was used in the study after additional validation, based on experimental data from the separation of CH_4_/CO_2_ mixtures. The model was fed with permeance coefficients of pure CH_4_, CO_2_, N_2_ and O_2_, which were experimentally determined in our laboratory in UMS-A5 and PRISM PA1020 modules.

## 2. Experimental Procedure and Mathematical Modelling

### 2.1. Experimental Setup

**Figure 1:**
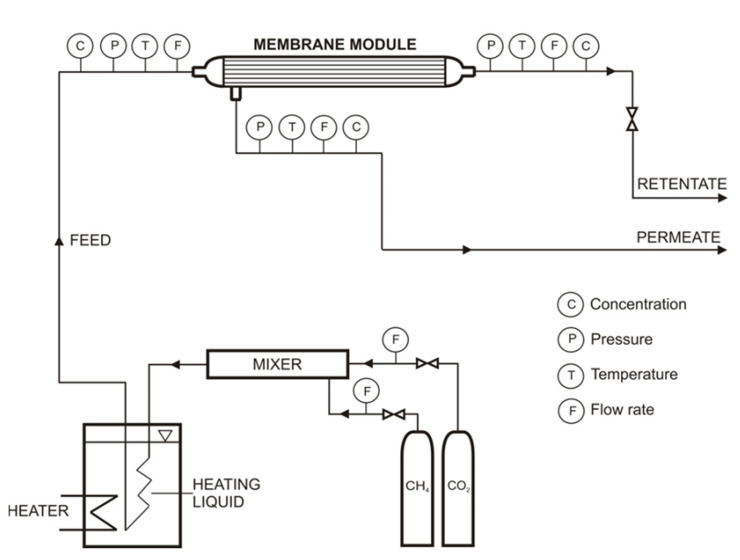
Experimental setup for gas permeation studies.

Gas permeation studies were performed in an experimental setup with the exchangeable membrane module, which is presented schematically in Figure 1. The setup is equipped with the gas preparation section, which consists of gas cylinders, a mixer and a thermostat. The gas from 1 or 2 gas cylinders passes through a mixer, which is a steel column with a diameter of 20 mm and a length of 500 mm. Its temperature was further established in the water thermostat (Julaba F12, the accuracy of temperature stabilization: 0.1 °C) before entering the membrane module. The module, the feed gas and the permeate and retentate lines, are insulated. Pressure, temperature, flow rate and composition (in the case of gas mixtures) were measured and recorded during experiments for the three gaseous streams. In the case of composition, the three-channel Varian (CP-4900) microchromatograph was used, thus the concentration of the mixture components was measured in the feed gas, permeate and retentate at the same time with an accuracy of 0.01 vol.%. The pressure was measured with pressure transducers (Cole-Parmer P series) with an accuracy of 0.1 psi. For the temperature measurement, Cole-Parmer Digi-Sense was used, which provided an accuracy of 0.1 °C. Aalborg GFM37 flow meters with an accuracy of 0.1 L min^−1^ were used to measure the feed gas, retentate and permeate flow rates.

### 2.2. Membrane Modules

Two hollow-fibre modules were used: PRISM PA1020, provided by Air Products, and UMS-A5, provided by UBE. The first one has a membrane formed of modified polysulfone and in the UBE’s module, a polyimide membrane was used. Both modules were designed mainly for air separation but were also successfully implemented in a hybrid, adsorptive membrane process for CO_2_ capture [32,33]. The modules were used in the experimental setup as supplied, and parameters used in the experiments were within the pressure and temperature ranges recommended by the manufacturers. The membrane area in these modules was 2.24 m^2^ in the case of PRISM PA1020 [33,34] and 0.167 m^2^ [35] in the case of UMS-A5. Other technical parameters of the modules were given in [29].

### 2.3. Gas Permeation Studies

Permeance coefficients of pure CO_2_, N_2_ and O_2_ in both modules were determined experimentally in our previous studies [34,35]. In the case of pure methane, the permeance was determined experimentally at 295 K. For this purpose, the feed gas pressure was changed within the range of 0.12–0.72 MPa (a) and the corresponding permeate flow rate was measured. The absolute permeate pressure was close to the ambient pressure, but was recorded for every single experimental point. The permeate flow rate is a basis for the determination of the gas permeance, as follows:

(1) $$Q=\frac{P_{\omega }}{\left(p_{F}-p_{P}\right)/A}$$

where *A*—membrane area (m^2^); *Q*—permeance—kmol h^−1^ MPa^−1^ or GPU; *P_ω_*—permeate flow rate (kmol h^−1^); *p_F_* and *p_P_*—feed pressure and permeate pressure, respectively (MPa).

The ideal separation factor (α) of carbon dioxide, nitrogen and oxygen vs. methane may be defined, in this case, as follows:

(2) $$\propto_{i/CH_{4}}=\frac{Q_{i}}{Q_{CH_{4}}}\;$$

Although *Q* is virtually independent of the feed gas flow rate, the permeation of pure gases was measured at two values of the feed gas flow rate of 0.025 and 0.038 kmol h^−1^ (PRISM PA1020), and at three values of the feed gas flow rate of 2.5 × 10^−4^, 5.0 × 10^−4^ and 7.5 × 10^−4^ kmol h^−1^ (UMS-A5), in order to check for experimental errors as well as for consistency of the setup and methodology.

The separation of two component mixtures containing 50 and 60 vol.% of methane in the mixture with carbon dioxide was investigated experimentally for the feed gas flow rate of 0.038 kmol h^−1^ (PRISM PA1020) and 2.5 × 10^−4^–9.6 × 10^−3^ kmol h^−1^ (UMS-A5), at the temperature of 294–296 K. The performance of the separation process was evaluated on the basis of CO_2_ concentration in the retentate and permeate, as well as the cut ratio, as follows:

(3) $$Cut\;ratio=\frac{P_{\omega }}{F_{\alpha }}\;$$

where *F_α_*—feed gas flow rate (kmol h^−1^). At a given pressure and flow rate, one experimental session consisted of the following steps: the establishment of parameters of feed gas, reaching the steady state and monitoring of the process in the steady state. The process parameters (i.e., pressure, flow rate, composition and temperature) of the feed gas, retentate and permeate were monitored and recorded during the entire experimental session. The recording of the concentrations was started every 5 min and it took about 4 min. Other parameters were recorded in a continuous way. The concentrations and flow rates reported in Section 3.2 are their average values determined in the steady state within a reasonable long time period (~100 min).

### 2.4. Mathematical Model

**Figure 2:**
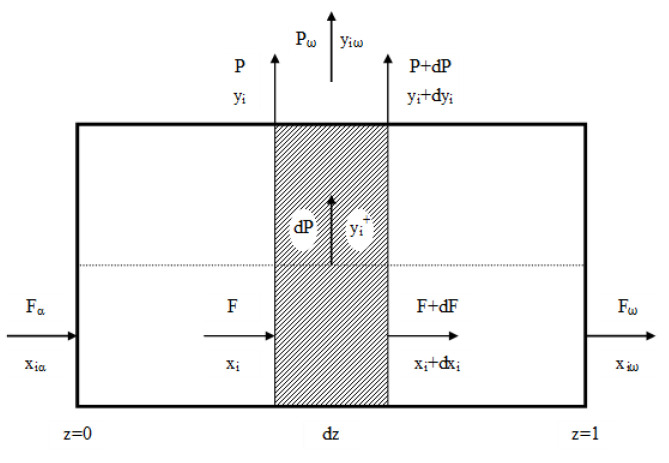
Gaseous streams in a membrane module for plug flow on the feed side and unhindered flow on the permeate side.

A gaseous mixture introduced to the membrane module is divided into two outlet streams: the permeate, which is the stream transported through the membrane, and the retentate, which is the stream remaining on the feed side of the module. The permeate may flow co-currently or counter-currently to the feed gas, or it may be locally unhindered [36]. Taking into account that the permeate is collected in the inter-tubular space and its flow is unforced, we assumed the plug flow on the feed side and unhindered flow on the permeate side in both modules. Such an attempt was successfully validated and used in our studies concerning membrane and hybrid processes of CO_2_ capture from mixture with nitrogen. The flow pattern is schematically presented in Figure 2.

It was also assumed that there are no interactions between the permeating components (therefore, the permeances are the same as for pure components), that the pressure drop and axial dispersion are negligible on both sides of the membrane, that the process is isothermal and that concentration polarization is also negligible on both sides of the membrane. The final forms of model equations and boundary conditions are presented in Table 1. All phenomena accompanying the transport of gas through the membrane are included in permeances, which may be measured directly, as described in the previous section.

The set of first-order ordinary differential equations with accompanying algebraic equations and boundary conditions, as presented in Table 1, was implemented and solved in a C++ numerical simulator. The Runge–Kutta method of the 4th order, with a given integration step *dz*, was used, and the integration started from the feed gas inlet and ended at the retentate outlet.

## 3. Results and Discussion

### 3.1. Permeability and Ideal Selectivity of Main Biogas Components

**Figure 3:**
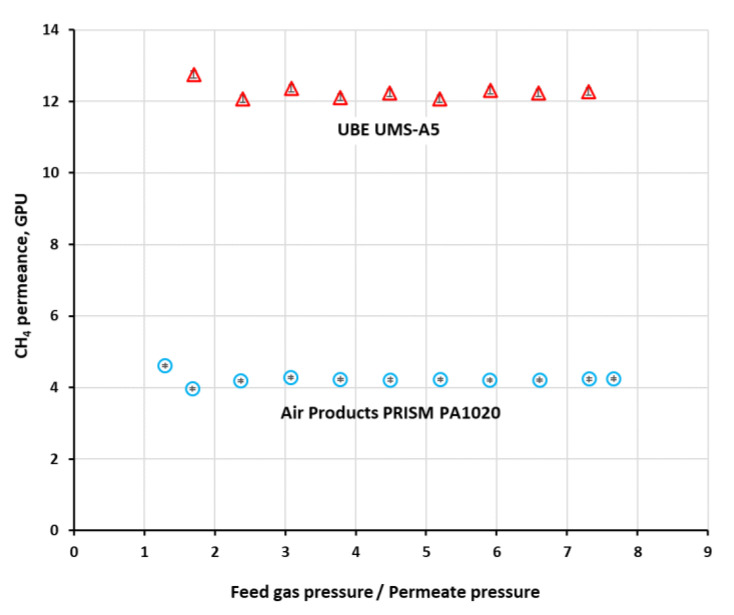
The permeance of CH_4_ against the feed to permeate pressure ratio in PRISM PA1020 (feed gas flow rate: 0.038 kmol h^−1^, 294.5 K) and UMS-A5 (feed gas flow rate: 7.5 × 10^−4^ kmol h^−1^, 296.4 K).

The permeance (*Q_i_*) of methane in both investigated modules, determined from single gas experiments, was shown graphically in Figure 3 for feed gas flow rate of 0.038 kmol h^−1^ in the case of PRISM PA1020 and 7.5 × 10^−4^ kmol h^−1^ in the case of UMS-A5. As can be seen in the figure, the permeance is virtually independent of pressure in the experimental conditions. Since it is independent of feed gas flow rate as well, presented in Table 2 are their average values from experimental data, concerning feed gas flow rates of 0.025 and 0.038 kmol h^−1^ in the case of PRISM PA1020 and 2.5 × 10^−4^, 5.0 × 10^−4^ and 7.5 × 10^−4^ kmol h^−1^ in the case of UMS-A5. The permeance coefficients of other main biogas components, i.e., carbon dioxide, nitrogen and oxygen, determined at 293 K in our previous studies, are also given in Table 2 as a reference.

The permeance of all gases in UMS-A5 with the polyimide membrane is significantly higher than that in PRISM PA1020 with the polysulfone one. More importantly, the ideal selectivity of CO_2_, O_2_ and N_2_ vs. methane was also visibly (2.4–2.8 times) higher in the UMS-A5 module. Thus, in terms of separation properties, the module with the polyimide membrane is expected to perform better. The permeance of CO_2_, N_2_ and O_2_ presented in Table 2 concerns fresh, unused earlier modules and was used in a simulation study, discussed in Section 3.4. In the case of the experimental study discussed in Section 3.3, both modules have been used in other separations for a few years and their separation properties have changed [34]. In this case, current permeance coefficients of CO_2_ were determined and used for the purpose of model validation, which were equal to 128.1 and 992.2 GPU in PRISM PA1020 and UMS-A5, respectively.

In the case of PRISM PA1020 the permeance coefficient of CH_4_ measured in our laboratory (4.2 GPU) as well as the ideal CO_2_/CH_4_ (36.4), O_2_/CH_4_ (6.55) and N_2_/CH_4_ (0.89) are selectivity in conformity with data given in [37] for the similar Air Products PRISM module. In the case of the UBE UMS-A5 module with polyimide, the permeance of methane was not previously reported, but that of CO_2_ and N_2_ as measured in our laboratory agrees well with data provided by [38], i.e., CO_2_ permeance of 1300 GPU and the ideal CO_2_/N_2_ selectivity of 41 at 298 K.

### 3.2. Separation of CH_4_/CO_2_ and Model Validation

**Figure 4:**
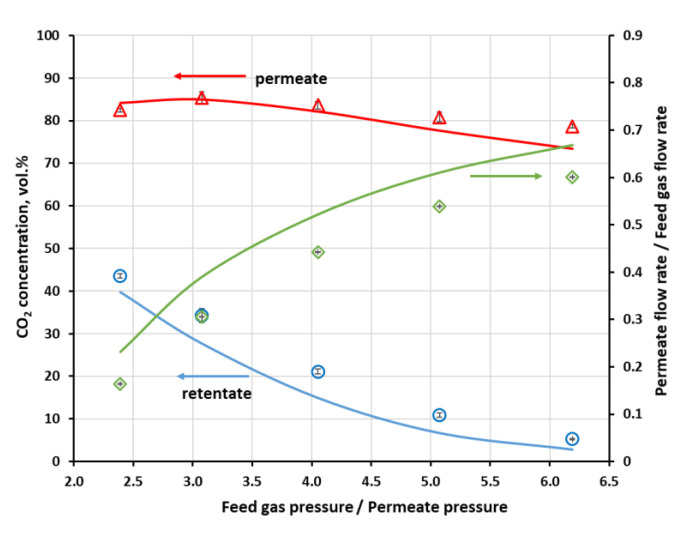
Experimental (points) and theoretical (lines) CO_2_ concentration in the retentate and permeate, along with flux cut ratio against feed to permeate pressure ratio in Air Products PRISM PA1020 module. Feed gas flow rate: 0.038 kmol h^−1^; composition of feed gas: CH_4_ (50 vol.%)/CO_2_ (50 vol.%); temperature: 295 K.

**Figure 5:**
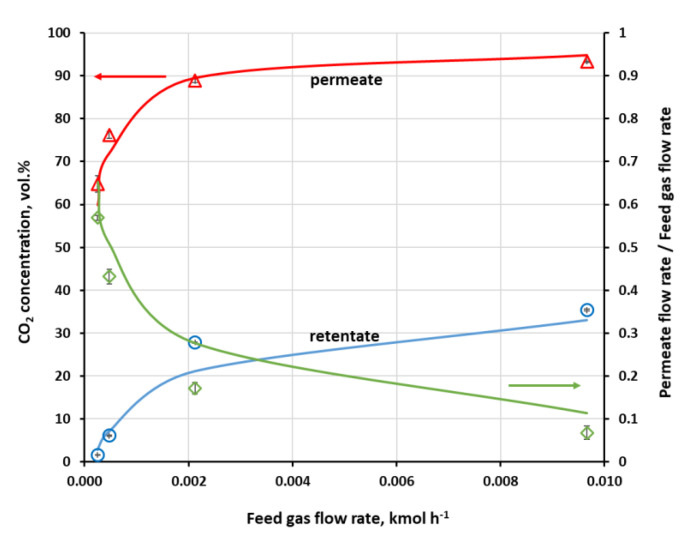
Experimental (points) and theoretical (lines) CO_2_ concentration in the retentate and permeate along with flux cut ratio against feed gas flow rate in UBE UMS-A5 module. Feed gas pressure: 0.4 MPa (a); composition of feed gas: CH_4_ (60 vol.%)/CO_2_ (40 vol.%); temperature: 295 K.

The separation of methane and carbon dioxide mixture was investigated in both modules for different CH_4_ concentrations (50 and 60 vol.%) at the module inlet, as well as for various feed gas flow rates (2.5 × 10^−4^–9.6 × 10^−3^ kmol h^−1^) in the case of UMS-A5. In such a situation, the main product is retentate, which is enriched in methane. However, in this section, the analysis was focused on carbon dioxide since this is the main undesired component in the raw biogas and the efficiency of its removal determines the general separation performance of the membrane biogas upgrading process. Experimentally measured, using a three-channel Varian microchromatograph, CO_2_ content in the retentate, permeate and flux cut ratio against the pressure ratio of the feed gas and permeate are presented in Figure 4 for Air Products PRISM PA1020. Each experimental point marked in Figure 4 and Figure 5 is the arithmetic mean determined from 10 measurement points. As can be seen in Figure 4, CO_2_ concentration in permeate passes through a maximum while the feed to permeate pressure ratio is increased. On the retentate side, carbon dioxide content is monotonically decreased with p_F_/p_P_ rise to 5.26 vol.% at the maximum p_F_/p_P_ value. Thus, for the current feed gas flow rate (0.038 kmol h^−1^) and CO_2_ content (50 vol.%), the feed to permeate pressure ratio would have to be raised above 5.7 in order to meet the requirements for carbon dioxide content in biomethane. However, this would be done at the cost of a further increase in the cut ratio (well above 0.6), i.e., a drop in the enriched methane stream.

On the other hand, CO_2_ content in retentate in the UMS-A5 module is well below 3 vol.% at the feed gas pressure of 0.4 MPa (a) and the lowest flow rate. In this paper, the authors use absolute pressure. This can be seen in Figure 5, where experimentally determined CO_2_ concentration in the retentate and permeate, along with flux cut ratio against feed gas flow rate, are presented for this module. The cut ratio is about 0.6 for this lowest feed gas flow rate. Carbon dioxide concentration in retentate raises quickly with the increase in the feed gas flow rate, especially in the region of its lower values. The module has a small membrane area; therefore, a moderate drop in contact time significantly lowers the amount of carbon dioxide transported through the membrane, despite rather high CO_2_ permeance.

While some conclusions presented in this section seem obvious, the experiments provided the necessary data to perform a validation of the mathematical model presented in Section 2.4. Results of numerical simulations, shown by lines in Figure 4 and Figure 5, fit well with the experimental data in the whole range of feed to pressure ratio, carbon dioxide content in the feed gas and gas flow rate at module inlet. The qualitative agreement between experimental and numerical data seems to be very good. From a quantitative point of view, the average relative error between experimental data and the results of simulations lies in the range of 1.9–3.9% and 14.3–30.2 in the case of CO_2_ concentration in permeate and retentate, respectively, and 14.5–40.1% in the case of cut ratio.

The more visible quantitative discrepancy between the model and experiments can be seen in the case of cut ratio, especially for UBE UMS-A5. As can be seen from Equations (13) and (14), the retentate and, consequently, the permeate flow rate, are derived from calculated CO_2_ mole fractions in these streams and thus depend directly on the accuracy with which they are determined. Taking into account that the model presented here reflects experimental conditions and was successfully validated in other separation cases, the problem probably lies in the fact that ideal instead of actual permeance coefficients were used. Both modules have glassy polymer membranes and, since the plasticization induced by carbon dioxide was not observed in the investigated modules [34], the competitive sorption in fractional free volume seems to be responsible for the varying change in permeances of both mixture components.

The problem was lately raised by Miandoab et al. [39] in their attempt concerning strict modelling of biogas upgrading in a module with a Matrimid membrane. They used the dual mode sorption (DMS) model in order to determine the concentration of gases in the glassy polymer. The required DMS parameters are usually not known or are not possible to determine in the case of the membranes that are used in commercial modules, as investigated in the current study. However, this problem should be taken into account in the development and optimization of the membrane biogas upgrade process, based on such modules. For the purpose of this study, the values of pure component permeance were further used, since a general agreement between experiments and simulations is still acceptable.

### 3.3. Background to the Simulation of a Biogas Upgrade Membrane Process

**Figure 6:**
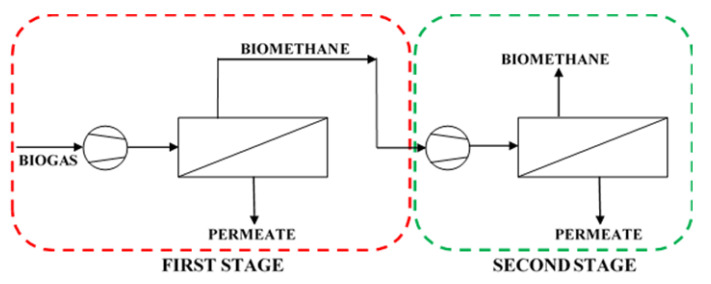
Scheme of the membrane biogas upgrading process.

Polysulfone and polyimide membranes used in Air Products PRISM PA1020 and UBE UMS-A5 modules, respectively, were assessed theoretically in the process of upgrading the stream of 5 m^3^ (STP) h^−1^ (0.223 kmol h^−1^) of pretreated biogas from an agricultural biogas plant in southern Poland. The membrane process analyzed in this section is presented schematically in Figure 6 and the basic parameters used in the simulation are given in Table 2 and Table 3. The mathematical model, presented in Section 2.4, was used in this analysis, which deals with two process options. In the first one, biogas is upgraded in a single stage, where the feed mixture is compressed and split into two gas streams. The biomethane leaves the module as retentate at the same pressure as the feed gas. In turn, the permeate stream is derived at the ambient pressure. This case is highlighted by a dashed red line in Figure 6. In the two-stage configuration a CH_4_-enriched stream from the first stage is directed after the compression to the second stage, from which the final biomethane stream is derived as retentate, along with the second waste permeate stream. In this configuration it was assumed that the same membrane is used in both stages. Pressure ranges applied in the simulations are in conformity with the requirements for the commercial modules investigated in this paper.

An assessment of the efficacy of a biogas upgrading process should be based, of course, on its economy. Such an analysis, which has to embrace also economic and/or process optimization, is beyond the scope of this paper. For the purpose of this study, the process was assessed in terms of the membrane productivity and the excess of thermal power carried with the biomethane stream, over the electric power necessary to carry out the essential separation process. The latter is a direct measure concerning the energetic efficiency of the essential separation process, but it also shows whether any profit from the production and sale of biomethane can be expected at all. It was defined as follows:

(15) $$Power\;excess=\frac{HHV_{CH_{4}}\cdot {\left[CH_{4}\right]}_{BM}\cdot {\dot{V}}_{BM}}{100\cdot \left(P_{s1}+P_{s2}\right)}$$

where *HHV_CH4_*—the heat of combustion (kWh_th_ m^−3^); *[CH4]_BM_*—methane concentration in biomethane (vol.%); ${\dot{V}}_{BM}$—biomethane flow rate (m^3^ (STP) h^−1^); *P_s1_* and *P_s2_*—compressor power in stage 1 and 2, respectively (kW_el_).

Parameters of biomethane in Equation (15) concern the retentate derived from the one-stage configuration or the second stage in the two-stage configuration. The power of the compressor may be expressed as:

(16) $$P_{si}=\frac{p_{n}\left(F_{si}^{in}\cdot 22.42/3600\right)\frac{\kappa }{\kappa -1}\left\{{\left(\frac{p_{si}^{out}}{p_{si}^{in}}\right)}^{\frac{\kappa -1}{\kappa }}-1\right\}}{1000*\eta }$$

where *p_n_*, *p_si_^in^* and *p_si_^out^*—pressure under standard condition, before and after the compressor in *“i”* stage, respectively (Pa); *κ*—heat capacity ratio; *F_s1_^in^*—flow rate before the compressor in *“i”* stage (kmol h^−1^); *η*—efficiency of the compressor.

Membrane productivity can serve as a rough indicator for the investment costs and can be defined as follows:

(17) $$Membrane\;productivity=\frac{HHV_{CH_{4}}\cdot {\left[CH_{4}\right]}_{BM}\cdot {\dot{V}}_{BM}}{100\cdot A}$$

Power excess index and membrane productivity are associated with specific energy and membrane area, respectively, which are reported in other studies. Indexes used in this study were also more convenient because the grid gas fuel consumption in Poland is billed on the basis of its heat of combustion.

The recovery of methane and carbon dioxide is defined below. In the two-stage configuration, the retentate parameters in Equation (18) concern the second stage and the permeate parameters in Equation (19) concern the first stage.

(18) $$Recovery\;CH_{4}=\frac{x_{\omega ,CH4}\;F_{\omega }}{x_{\alpha ,CH4}\;F_{\alpha }}\;$$

(19) $$Recovery\;CO_{2}=\frac{y_{\omega ,CO2}\;P_{\omega }}{x_{\alpha ,CO2}\;F_{\alpha }}\;$$

### 3.4. Biogas Enrichment in One-Stage Configuration

**Figure 7:**
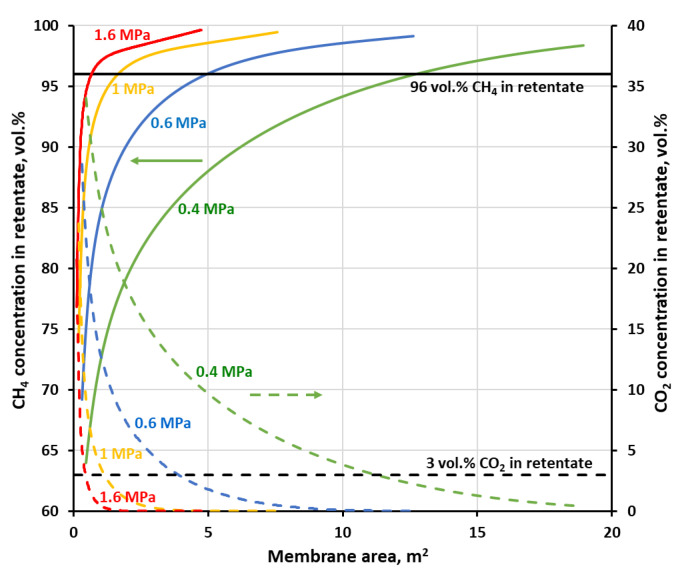
CH_4_ (solid lines) and CO_2_ (dashed lines) concentration in retentate against membrane area and feed gas pressure for a one-stage biogas upgrading system with the polyimide (UMS-A5) membrane.

**Figure 8:**
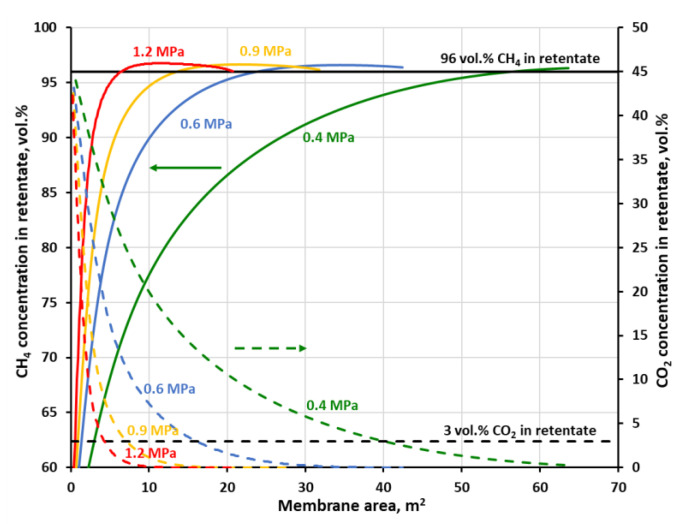
CH_4_ (solid lines) and CO_2_ (dashed lines) concentration in retentate against membrane area and feed gas pressure for a one-stage biogas upgrading system with the polysulfone (PRISM PA1020) membrane.

Figure 7 and Figure 8 show methane and carbon dioxide concentrations in retentate as a function of membrane area at the different feed gas pressures, when the separation of the four-component biogas is performed in the one-stage membrane configuration. As may be seen in the figures, in every case when CH_4_ concentration reaches 96 vol.%, i.e., the required level for combustible components in biomethane, the content of carbon dioxide has already been dropped below the required threshold of 3 vol.%. The other components of the biogas, nitrogen and oxygen have inlet concentrations below its threshold (4 and 0.1 vol.%, respectively). Oxygen permeates significantly faster than methane and nitrogen in both membrane materials and quickly passes to the permeate side. Its concentration was not shown in figures for the sake of their improved clarity, neither was the nitrogen content. Although, the latter is increased slightly during the separation but was always well below 4 vol.%.

As expected, an increase in the feed gas pressure leads to the drop in the membrane area required to achieve the separation goal, which is unambiguously determined in this case by a line of 96 vol.% of CH_4_ in Figure 7 and Figure 8. As can be seen in these figures, the concentration profiles become sharper when the pressure of the feed gas is raised. Therefore, again related to the lowering of the membrane area, it is more pronounced in the low pressure region. Thus, in the case of polyimide membrane (UMS-A5), the required membrane area is significantly reduced (from 12.7 to 1.7 m^2^) while raising the feed gas pressure from 0.4 to 1 MPa. However, after a further increase in pressure from 1 to 1.6 MPa, the membrane area drops only to 0.7 m^2^. The same effect is observed for polysulfone membrane used in PRISM PA1020, except that the required membrane area is much higher and is equal to 56.7, 13.6 and 0.6 m^2^ for a pressure of 0.4, 0.8 and 1.2 MPa, respectively. The other difference between the two membrane materials is the shape of the concentration profile of methane in the retentate. In the case of polyimide from UMS-A5, CH_4_ content in retentate continues to rise monotonically with an increase in membrane area after passing the 96 vol.% thresholds. On the other hand, in polysulfone membrane, the methane concentration passes through this threshold and, after reaching a maximum, decreases with a further increase in membrane area, which is the sign that a significant amount of this gas is derived from the product to the permeate. This lower general separation performance of the polysulfone membrane, in comparison with the polyimide one, follows from lower permeance of components, which are accompanying methane in biogas, and lower ideal selectivity, as was shown in Table 2.

**Figure 9:**
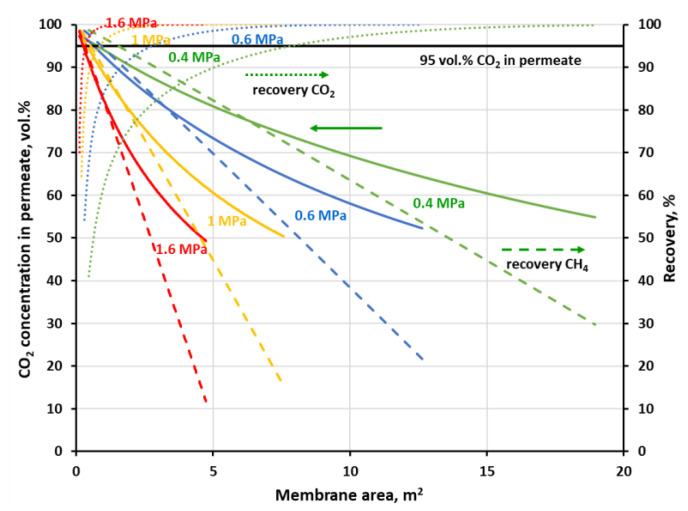
CO_2_ concentration (solid lines) and recovery (dotted lines) in permeate along with CH_4_ recovery (dashed lines) in retentate against membrane area and feed gas pressure for a one-stage biogas upgrading system with the polyimide (UBE UMS-A5) membrane.

**Figure 10:**
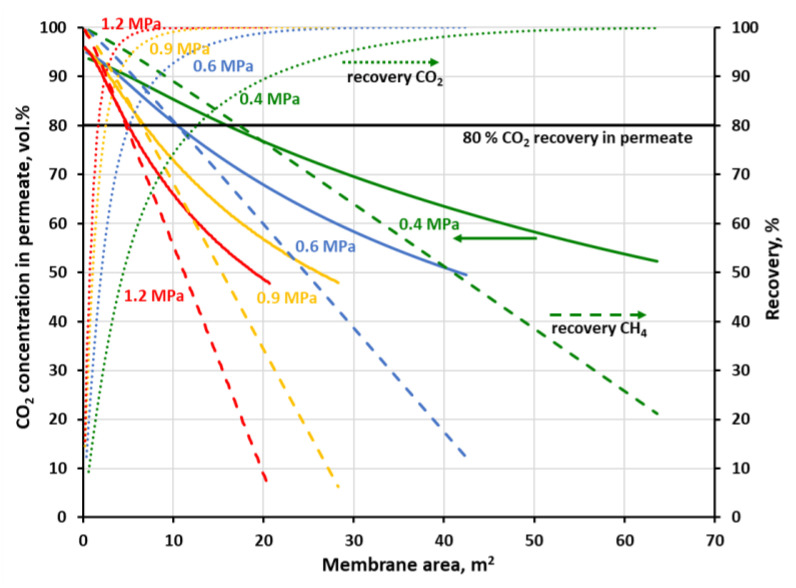
CO_2_ concentration (solid lines) and recovery (dotted lines) in permeate along with CH_4_ recovery (dashed lines) in retentate against membrane area and feed gas pressure for a one-stage biogas upgrading system with the polysulfone (PRISM PA1020) membrane.

The recovery of methane which is associated with 96 vol.% of CH_4_ on the concentration profiles in Figure 7 and Figure 8 is equal to 53.4, 82.5 and 88.6% at 0.4, 1 and 1.6 MPa, respectively, for the polyimide membrane and 30, 61.8 and 71.6% at 0.4, 0.8 and 1.2 MPa, respectively, for the polysulfone one. At the highest pressures, this recovery is in line with theoretical data concerning the trade-off between CH_4_ purity and CH_4_ recovery, reported by Scholz et al. [19] for the separation of 50% CO_2_ and 50% CH_4_. Methane recovery is presented in Figure 9 and Figure 10 as a function of membrane area at the different feed gas pressure along with carbon dioxide concentration and recovery in permeate. The values of CH_4_ recovery at higher pressures are thus acceptable in the case of the one-stage system based on the polysulfone membrane, and even very good for the polyimide membrane. Thus, it can be stated that an upgrade of this particular biogas to biomethane is possible in a one-stage membrane process based on UMS-A5 or PRISM PA1020 module types. However, as is discussed in detail in the following section, a high methane recovery is not necessarily accompanied by a favorable relationship between thermal power, carried with the biomethane stream, and the electric power necessary to carry out the essential separation process.

Carbon dioxide in the feed biogas is a ballast, which should be compressed before separation, and the main component of permeate—the waste stream from the process. Therefore, the general efficacy of a membrane biogas upgrade process is closely related to the effectiveness of the transport of large amounts of CO_2_ through the membrane. From this point of view, the biogas upgrading process may also be associated with carbon dioxide capture [10]. Since carbon dioxide has the highest permeance and CH_4_/CO_2_ ideal selectivity, it can be removed to a large degree in the first stage of a two-stage process at the lower feed gas pressure. The separation goal in such a first stage may be focused on the obtaining of a CO_2_-rich stream for further treatment and utilization or a high CO_2_ recovery. As it was shown in Figure 9, when the goal is on 95 vol.% of the CO_2_ purity in permeate, 55% of carbon dioxide could be initially removed in a stage with the UBE polyimide membrane of 0.76 m^2^. When the pressure is increased to 1 MPa, the CO_2_ recovery is raised to 86.4% and the required membrane area drops to 0.39 m^2^. At the same time, 97.7% (at 0.4 MPa) and 96.4% (at 1 MPa) of methane may be recovered in the retentate stream and further enriched in the next membrane stage. In Figure 10 was shown a case when 80% of carbon dioxide is to be recovered in a one-stage system with Air Products polysulfone membrane. In this case, purity of 82.8 vol.% CO_2_ in permeate can be achieved at 0.4 MPa and the membrane area of 12.7 m^2^. When raising the feed gas pressure to 0.8 MPa, the CO_2_ purity is increased to 90.2 vol.% and the membrane area is decreased to 3.2 m^2^. The corresponding methane recovery in the retentate stream is equal to 85.7 and 92.4% at 0.4 and 0.8 MPa, respectively.

### 3.5. Biogas Enrichment in Two-Stage Configuration and Its Energy Efficiency

In a two-stage configuration considered in this paper, the CH_4_-enriched stream derived as retentate from the first stage is fed to the second membrane stage after a further compression (cf. Figure 6). It was assumed that feed gas pressure (and, accordingly, the retentate pressure) in the first stage was equal to 0.4, 0.6 or 0.8 MPa. The pressure of feed gas at the inlet to the second membrane stage was further increased (up to 1.6 MPa in the case of the module with UBE polyimide membrane and up to 1.2 MPa in the case of the module with Air Products polysulfone membrane). As mentioned above, in the first membrane stage, the majority of carbon dioxide amount is intended to be removed from the biogas. Thus, in the case of polyimide membrane, it was assumed that CO_2_ concentration in permeate derived from the first stage should be at least 95 vol.% at the given flow rate, pressure and composition of biogas as well as the membrane area. The latter was determined in the single-stage simulations discussed in the previous section. For the second membrane material, it was assumed that 80% of CO_2_ is recovered from biogas in the first stage. The parameters concerning this stage (pressure, membrane area, the composition of retentate and permeate) were summarized in Table 4. Retentate composition and flow rate in the table are the inlet gas parameters for stage 2.

**Figure 11:**
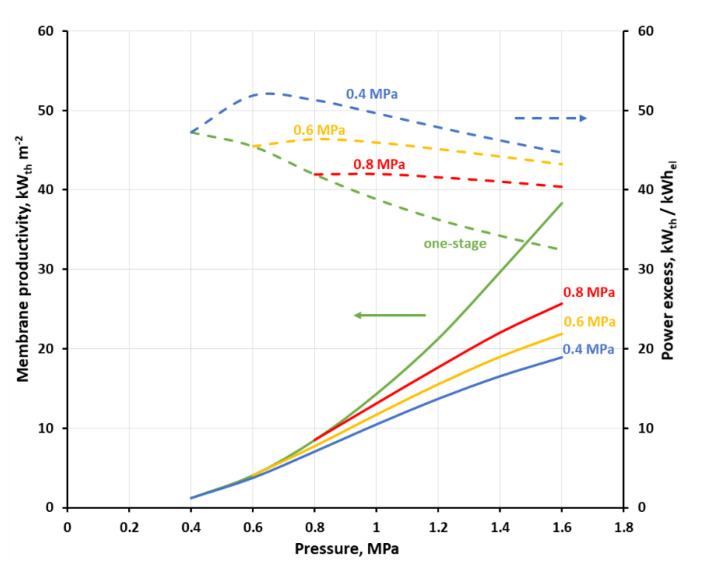
Membrane productivity (solid lines) and power excess index (dashed lines) as a function of gas pressure on the feed side for one-stage configuration and two-stage configuration at feed gas pressure of 0.4, 0.6 and 0.8 MPa in the first stage inlet when polyimide (UMS-A5) membrane is used.

**Figure 12:**
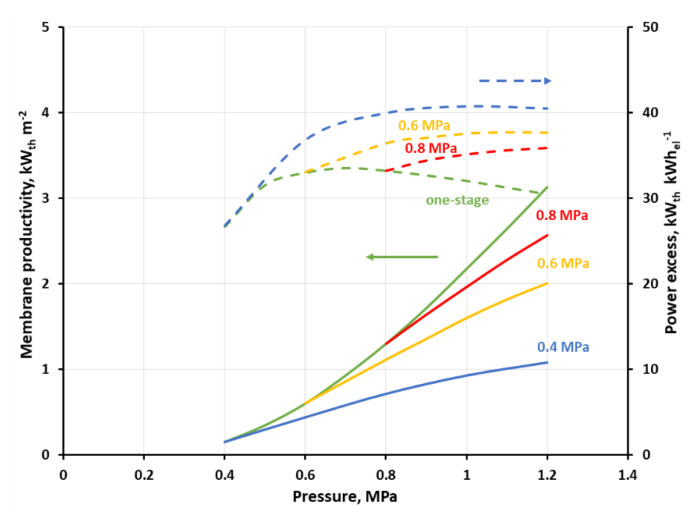
Membrane productivity (solid lines) and power excess index (dashed lines) as a function of gas pressure on the feed side for one-stage configuration and two-stage configuration at feed gas pressure of 0.4, 0.6 and 0.8 MPa in the first stage inlet when polysulfone (PRISM PA1020) membrane is used.

All simulation cases were summarized in Tables S1–S8 in the supplementary material. Figure 11 and Figure 12 show the membrane productivity and power excess index as a function of inlet gas pressure to stage 2 at the different feed gas pressure in stage 1, when CH_4_ concentration in retentate derived from stage 2 reaches 96 vol.%. As it was explained earlier, in this particular separation case, it means that the concentrations of other mixture components are below their respective thresholds required for biomethane. The appropriate results for the single-stage system are represented by green lines and serve as a reference. As can be seen in the figures, the membrane productivity drops when the process is split into two stages. The difference in this indicator, as compared to the single-stage system, is the highest when the pressure in the first stage (p_s1_) is equal to 0.4 MPa, and in a general case is also increased, with the rise in the pressure in the second stage. When the feed gas pressure in the first stage is increased, the membrane productivity tends to be closer to that of the single-stage system. An adverse influence of the process splitting into two stages on the membrane productivity is much more pronounced when the separation in the first stage is focused on a high CO_2_ recovery (cf. Figure 12). In this case, the membrane productivity is generally an order of magnitude smaller, compared with that presented in Figure 11, due to the lower separation performance (in terms of permeance and ideal selectivity) of the polysulfone membrane in comparison with the polyimide one.

This reported drop in the membrane productivity is due to the fact that the membrane area required for the production of biogas which is in conformity with the country’s regulations becomes higher in this separation case when an additional stage is introduced. Additionally, a drop in methane recovery, and hence in the biomethane stream, was observed in the two-stage system. Therefore, in the case presented in Figure 11 the recovery changes in the range of 53.4–88.6% and 53.4–87.5% in the one- and two-stage configuration, respectively, within the pressure range of 0.4–1.6 MPa. In the second case (cf. Figure 12), the recovery varies in the range of 30.1–71.6% and 30.3–65% in the one- and two-stage configurations, respectively, within the pressure range of 0.4–1.2 MPa (cf. Tables S1–S8 in the supplementary material). The membrane productivity used in this study translates in a simple way to membrane specific area. Therefore, for example, the membrane productivity of 4.06–38.33 kW_th_ m^−2^ (UMS-A5, one-stage, 0.6–1.6 MPa) is equivalent to 1.25–0.28 m^2^ h m^−3^ (STP) of membrane specific area, as referred to biomethane flow rate. This is in line with data reported by Scholz et al. (1.57–1.7 m^2^ h m^−3^ (STP)) for the system with a much higher biogas flow rate of 1000 m^3^ (STP) h^−1^ [19].

A real benefit of introducing a simple two-stage system for this particular case of small-scale separation system lies in the increase in the heat power in the stream of biomethane as related to the total power required for gas compression in the process. As can be seen in Figure 11, power excess index (green dashed line) in the one-stage system, based on a polyimide UBE membrane, is monotonically decreasing from 47.2 to 32.4 kW_th_ kW_el_^−1^ with an increase in the feed gas pressure. In the two-stage system and at the inlet biogas pressure of 0.4 MPa this index is initially raising with the inlet gas pressure raise in stage two, then passes through a maximum (51.3 kW_th_ kW_el_^−1^) at p_s2_ = 0.6 MPa and is decreasing after a further pressure raise. The relative difference between the power excess index in the single and two-stage system raises from 14.1% at 0.6 MPa to 37.9% at 1.6 MPa. The course of the power excess lines is similar at p_s1_ of 0.6 and 0.8 MPa, i.e a maximum is observed, however the difference between two- and one-stage configuration becomes smaller when p_s1_ is increased. The power excess maxima can also be seen in Figure 12 at p_s1_ of 0.4 and 0.6 MPa, but they are moved towards higher p_s2_ pressures, equal, respectively to 1 and 1.1 MPa. The highest value of the power excess index, as seen in Figure 12 (40.7 kW_th_ kW_el_^−1^ at p_s1_ = 0.4 MPa and p_s2_ = 1 MPa), is smaller than in the other case, mainly due to the lower flow rate of biomethane, which is derived in this case. When the separation in the first stage is focused on a high CO_2_ recovery, this leads to a drop in methane recovery in this stage (cf. Table 4) and consequently in the entire two-stage system, which is only strengthened by the lower ideal CH_4_/CO_2_ selectivity in a polysulfone membrane, as compared with the polyimide one.

In this particular separation case, and the configuration with polyimide membrane, we focused in the first stage on a high CO_2_ purity (Figure 11); the highest efficacy from an energetic point of view is reached when the membrane productivity is rather moderate (7.06 kW_th_ m^−2^ at 0.6 MPa, two stages) and there is no significant difference between the one-stage system and the two-stage system. For the lowest power excess index (32.4 kW_th_ kW_el_^−1^ at 1.6 MPa) the highest membrane productivity is also reported (38.3 kW_th_ m^−2^), and both values concern the one-stage configuration. In the case of polyimide UBE membrane, the limiting values of the power excess index of 32.4 kW_th_ kW_el_^−1^ and 51.3 kW_th_ kW_el_^−1^ translate to the specific energy of 0.33 and 0.21 kWh_el_ m^−3^ (STP), respectively. This corresponds well to data reported by Scholz et al. (0.28–0.32 kWh_el_ m^−3^ (STP)) for the system with the biogas flow rate of 1000 m^3^ (STP) h^−1^ [19] and by Struk et al. (0.14–0.26 kWh_el_ m^−3^ (STP)) as a general index for membrane biogas upgrading processes [8].

It seems that the indicators discussed above are high enough to think about the profitability of investing in a small-scale membrane installation for the production of biomethane. Making a very rough costs estimation should make this issue more clear. In terms of current unit costs of gas fuel “E” (0.0245 euro kWh_th_^−1^) and electricity (0.1373 euro kWh_el_^−1^) for households in Poland, the power excess index translates to 9.15 (=51.3 × 0.0245/0.1373) and 5.78 (=32.4 × 0.0245/0.1373) for its two limiting cases, presented in Figure 11. Thus, one can expect that the investment in such a membrane unit for the biogas upgrade should be beneficial from the point of view of exploitation cost. The same may be true in the configuration with polysulfone membrane and focused on the high CO_2_ recovery in the first stage (cf. Figure 12), where the power excess index, as expressed in the unit costs of energy carriers, varies at 1.2 MPa from 5.42 (one-stage system) to 7.23 (two-stage system, p_s1_ = 0.4 MPa). On the other hand, one can assess a prospective yearly economic profit from the unit area of membranes, based on the membrane productivity, which may serve as a reference concerning a ceiling for annual investment costs. Assuming that the operating time of the membrane upgrade installation during the year is 8000 h and in the configuration with polyimide membrane, we obtain 1383.8 euro m^−2^ year^−1^ (=7.06 × 8000 × 0.0245) at 0.6 MPa in two stages (cf. Figure 11), and 7506.8 euro m^−2^ year^−1^ (=38.3 × 8000 × 0.0245) at 1.6 MPa in one stage. In the configuration with polysulfone membranes, the expected membrane productivity was smaller, with 3.13 and 1.08 kW_th_ m^−2^ at 1.2 MPa, for one and two-stage system, which leads to 613.5 and 211.7 euro m^−2^ year^−1^, respectively. It is difficult to assess how it relates to the current cost of the membrane module layout. It seems, however, that there is a chance, especially in the case of a system build on polysulfone (UBE UMS-A5) membrane, for a return on investment in a reasonable payback period and economic income.

## 4. Conclusions

The upgrading of biogas from a small (5 m^3^ (STP) h^−1^) agricultural source in southern Poland into biomethane in membrane processes, based on membranes made from modified polysulfone and polyimide, available in the market in Air Products PRISM PA1020 and UBE UMS-A5 modules, respectively, was comprehensively investigated. It was theoretically shown that this is possible to obtain, in such a process, the stream of at least 96 vol.% of CH_4_ purity with the concentration of the other biogas components below their respective thresholds, as required in Poland for gas fuel “E”, with methane recovery of up to 87.5% and 71.6% for polyimide (UMS-A5) and polysulfone (PA1020), respectively. It was found that both membrane materials are comparable in terms of power excess index, which was as high as 51.3 kW_th_ kW_el_^−1^ and 40.7 kW_th_ kW_el_^−1^ in the case of polyimide and polysulfone, respectively. In turn, the membrane productivity was significantly higher in configurations with the polyimide (UMS-A5) membrane (up to 38.3 kW_th_ m^−2^ at 1.6 MPa with one stage) than those based on polysulfone (PA1020) membrane (up to 3.13 kW_th_ m^−2^ at 1.2 MPa with one stage).

The mathematical model, which was used in the analysis, has been validated against experimental data concerning the separation of CH_4_/CO_2_ in the PA1020 and UMS-A5 modules, with a satisfactory general agreement between the experiment and simulations. It was found, however, that in this particular separation case the competitive sorption in fractional free volume may lead to the change in permeances of both mixture components, which should be taken into account in the strict design and optimization of a membrane biogas upgrade process, based on membranes, used in UMS-A5 or PA1020 modules.

As a result of a rough costs estimation of a small-scale, membrane installation for the production of biomethane it was concluded that there is a chance, especially in the case of a system build on polysulfone (UBE UMS-A5) membrane, for a return on investment in a reasonable payback period and economic income. However, a strict economic analysis is required, which will embrace, among others, such issues as biogas pretreatment before basic membrane separation, biomethane utilization (sale to gas fuel “E” network and/or internal consumption), second product utilization (CO_2_-rich gas), a trade-off between investment costs and energy consumption, final system configuration (one or two-stage), and level of support for investors. This is, however, an independent and wide research topic that goes beyond the goal and volume of this study. We would like to address it in a separate study, which will be communicated in due course.

## Figures and Tables

**Table 1:** Mathematical model equations and boundary conditions.

Local mole fraction of component 1 on the permeate side (*y_1_^+^*):	
$y_{1}^{+}\overset{N}{\underset{i=1}{\sum }}\frac{\alpha_{i1}x_{i}}{x_{1}-\delta y_{1}^{+}\left(1-\alpha_{i1}\right)}-1=0$	(4)
where *δ*—pressure ratio (=p_P_/p_F_); *x*—mole fraction on the feed side; *N*—number of components. Local mole fractions (*y_i_^+^*) of the other permeate components (*i* = 2, …, *N*):	
$y_{i}^{+}=\frac{\alpha_{i1}x_{i}y_{1}^{+}}{x_{1}-\delta y_{1}^{+}\left(1-\alpha_{i1}\right)}$	(5)
Gas composition on the feed side (*i* = 1, …, *N* - 1):	
$\frac{dx_{i}}{dz}=-R\frac{y_{1}-x_{1}}{y_{1}-x_{1\alpha }}\left[\alpha_{i1}\left(x_{i}-\delta y_{i}^{+}\right)-x_{i}\overset{N}{\underset{j=1}{\sum }}\alpha_{j1}\left(x_{j}-\delta y_{j}^{+}\right)\right]$	(6)
where *R*—permeation number (=AQ_1_p_F_/F_α_); *x_α_*—mole fraction in the feed gas; *y*—mole fraction on the permeate side. Mole fraction of component N on the feed side:	
$x_{N}=1-\overset{N-1}{\underset{i=1}{\sum }}x_{i}$	(7)
Mole fraction of component 1 in the permeate:	
$\frac{dy_{1}}{dz}=R\frac{y_{1}-x_{1}}{x_{1\alpha }-x_{1}}\left[\left(x_{1}-\delta y_{1}^{+}\right)-y_{1}\overset{N}{\underset{j=1}{\sum }}\alpha_{j1}\left(x_{j}-\delta y_{j}^{+}\right)\right]$	(8)
Mole fractions of the other permeate components (*i* = 2, …, *N*-1):	
$y_{i}=\frac{x_{i}\left(y_{1}-x_{1\alpha }\right)-x_{i\alpha }\left(y_{1}-x_{1}\right)}{x_{1}-x_{1\alpha }}$	(9)
Mole fraction of component N on the permeate side:	
$y_{N}=1-\overset{N-1}{\underset{i=1}{\sum }}y_{i}$	(10)
Boundary conditions at *z* = 0 (*i* = 1, …, *N*):	
$x_{i}=x_{i\alpha }$	(11)
$y_{i}=y_{i\alpha }^{+}$	(12)
Retentate flow rate (*F_ω_*):	
$F_{\omega }=F_{\alpha }\frac{y_{1\omega }-x_{1\alpha }}{y_{1\omega }-x_{1\omega }}$	(13)
where *x_1α_*—mole fraction of component 1 in the feed gas; *x_1ω_*—mole fraction of component 1 in the retentate; *y_1ω_*—mole fraction of component 1 in the permeate. Permeate flow rate:	
$P_{\omega }=F_{\alpha }-F_{\omega }$	(14)

**Table 2:** The permeance of methane, carbon dioxide, oxygen and nitrogen [34,35].

	PRISM PA1020		UMS-A5	
Gas	Q, GPU	α_i/CH4_	Q, GPU	α_i/CH4_
**CH_4_**	4.20 ± 0.03	1	12.21 ± 0.11	1
**CO_2_**	152.77	36.4	1221.56	100.0
**O_2_**	27.5	6.55	227.54	18.6
**N_2_**	3.75	0.89	26.35	2.16

**Table 3:** Parameters used in simulations.

***Biogas***	
T, K	293
p_s1_^in^, MPa	0.1
F_α(s1)_ (=F_s1_^in^), kmol h^−1^	0.223
[CH_4_], vol.%	52
[CO_2_], vol.%	46.3
[O_2_], vol.%	0.1
[N_2_], vol.%	1.6
***Membrane unit***	0.1
p_P_, MPa	0.4–(1.2)1.6
p_F(s1)_ (=p_s1_^out^), MPa	0.4, 0.6, 0.8
p_s2_^in^, MPa	0.4–(1.2)1.6
p_F(s2)_ (=p_s2_^out^), MPa	
***Compressor power***	
κ	1.337
η	0.72

**Table 4:** Parameters in the first stage of the two-stage membrane system, when CO_2_ concentration in this stage is 95 vol.% (polyimide membrane) or CO_2_ recovery in this stage is 80% (polysulfone membrane).

**Polyimide Membrane (UBE, UMS-A5)**									
p_F_	A	Retentate						Permeate	
		F_ω_∙22.42	[CO_2_]	[O_2_]	[N_2_]	[CH_4_]	Recovery (CH_4_)	[CO_2_]	Recovery (CO_2_)
MPa	m^2^	m^3^ (STP) h^−1^	vol.%	vol.%	vol.%	vol.%	%	vol.%	%
0.4	0.76	3.66	28.44	0.1	2.08	69.38	97.68	95.1	55.03
0.6	0.63	3.16	17.94	0.1	2.36	79.6	96.77	95.03	75.51
0.8	0.49	2.98	13.32	0.1	2.49	84.1	96.47	95.06	82.85
**Polysulfone Membrane (Air Products, PRISM PA1020)**									
p_F_	A	Retentate						Permeate	
		F_ω_∙22.42	[CO_2_]	[O_2_]	[N_2_]	[CH_4_]	Recovery (CH_4_)	[CO_2_]	Recovery (CO_2_)
MPa	m^2^	m^3^ (STP) h^−1^	vol.%	vol.%	vol.%	vol.%	%	vol.%	%
0.4	12.73	2.76	16.58	0.09	2.53	80.81	85.68	82.83	80.26
0.6	5.25	2.89	15.6	0.1	2.54	81.76	90.75	88.22	80.55
0.8	3.18	2.92	15.15	0.11	2.55	82.2	92.46	90.2	80.87

## Data Availability

The data presented in this study are available on request from the corresponding author and the attached supplementary material.

## Supplementary Materials

The following are available online at https://www.mdpi.com/article/10.3390/membranes11120938/s1, Table S1: Results of the simulation of the biogas one-stage upgrade process with the polyimide membrane (UBE, UMS-A5), Table S2: Results of the simulation of the biogas two-stage upgrade process with the polyimide membrane (UBE, UMS-A5) and the pressure in the first stage of 0.4 MPa, Table S3: Results of the simulation of the biogas two-stage upgrade process with the polyimide membrane (UBE, UMS-A5) and the pressure in the first stage of 0.6 MPa, Table S4: Results of the simulation of the biogas two-stage upgrade process with the polyimide membrane (UBE, UMS-A5) and the pressure in the first stage of 0.8 MPa, Table S5: Results of the simulation of the biogas one-stage upgrade process with the polysulfone membrane (Air Products, PRISM PA1020), Table S6: Results of the simulation of the biogas two-stage upgrade process with the polysulfone membrane (Air Products, PRISM PA1020) and the pressure in the first stage of 0.4 MPa, Table S7: Results of the simulation of the biogas two-stage upgrade process with the polysulfone membrane (Air Products, PRISM PA1020) and the pressure in the first stage of 0.6 MPa, Table S8: Results of the simulation of the biogas two-stage upgrade process with the polysulfone membrane (Air Products, PRISM PA1020) and the pressure in the first stage of 0.8 MPa.
