# Supplementary material for: Upgrading Biogas from Small Agricultural Sources into Biomethane by Membrane Separation
Source: Membranes (Basel). 2021 Nov 27;11(12):938. doi: 10.3390/membranes11120938 (PMC8706255; doi:10.3390/membranes11120938)
Supplement: Supplementary file 1 [file membranes-11-00938-s001.zip › membranes-1474952-supplementary.pdf]

membranes

Supplementary Materials: Upgrading Biogas from Small Agricultural Sources into Biomethane by Membrane Separation

Aleksandra Janusz-Cygan , Jolanta Jaschik and Marek Tańczyk

Table S1. Results of the simulation of the biogas one-stage upgrade process with the polyimide membrane (UBE, UMS-A5).

p_F	A	V_{BM}	[CH_4]	Recovery (CH_4)	P_{S1}	P_{S2}	P_{BM}	Power excess	Membrane productivity	Specific energy	Membrane specific area
MPa	m^2	m^3 (STP) h^{-1}	vol.%	%	kW_{el}	kW_{el}	kW_{th}	$kW_{th}kW_{el}^{-1}$	kW_{th} m^{-2}	kW_{el} m^{-3} (STP)	m^2 h m^{-3} (STP)
0.4	12.70	1.45	96.02	53.38	0.32	0	15.32	15.32	1.21	0.22	8.78
0.6	4.95	1.90	96.02	70.13	0.44	0	20.13	20.13	4.06	0.23	2.61
0.8	2.63	2.11	96.02	78.03	0.53	0	22.40	22.40	8.50	0.25	1.25
1.0	1.65	2.23	96.03	82.47	0.61	0	23.67	23.67	14.32	0.27	0.74
1.2	1.15	2.31	96.04	85.24	0.68	0	24.46	24.46	21.28	0.29	0.50
1.4	0.84	2.36	96.00	87.30	0.73	0	25.06	25.06	29.67	0.31	0.36
1.6	0.66	2.40	96.03	88.58	0.78	0	25.42	25.42	38.33	0.33	0.28

Table S2. Results of the simulation of the biogas two-stage upgrade process with the polyimide membrane (UBE, UMS-A5) and the pressure in the first stage of the 0.4 MPa.

p_F	A	V_{BM}	[CH_4]	Recovery (CH_4)	P_{S1}	P_{S2}	P_{BM}	Power excess	Membrane productivity	Specific energy	Membrane specific area
MPa	m^2	m^3 (STP) h^{-1}	vol.%	%	kW_{el}	kW_{el}	kW_{th}	$kW_{th}kW_{el}^{-1}$	kW_{th} m^{-2}	kW_{el} m^{-3} (STP)	m^2 h m^{-3} (STP)
0.4	12.69	1.45	96.01	53.42	0.32	0	15.33	47.28	1.21	0.22	8.77
0.6	5.33	1.89	96.01	69.68	0.32	0.06	20.00	51.89	3.75	0.20	2.82
0.8	3.14	2.09	96.01	77.35	0.32	0.11	22.20	51.31	7.06	0.21	1.50
1.0	2.23	2.21	96.02	81.61	0.32	0.15	23.42	49.65	10.49	0.21	1.01
1.2	1.77	2.28	96.02	84.34	0.32	0.18	24.21	47.89	13.71	0.22	0.77
1.4	1.50	2.33	96.01	86.21	0.32	0.21	24.74	46.24	16.55	0.23	0.64

1.6	1.33	2.37	96.02	87.52	0.32	0.24	25.12	44.71	18.93	0.24	0.56

Table S3. Results of the simulation of the biogas two-stage upgrade process with the polyimide membrane (UBE, UMS-A5) and the pressure in the first stage of 0.6 MPa.

p_F	A	V_{BM}	$[CH_4]$	Recovery (CH_4)	P_{S1}	P_{S2}	P_{BM}	Power excess	Membrane productivity	Specific energy	Membrane specific area
MPa	m^2	m^3 (STP) h^{-1}	vol.%	%	kW_{el}	kW_{el}	kW_{th}	$kW_{th}kW_{el}^{-1}$	kW_{th} m^{-2}	kW_{el} m^{-3} (STP)	m^2 h m^{-3} (STP)
0.6	4.94	1.90	96.01	70.21	0.44	0	20.15	45.52	4.08	0.23	2.60
0.8	2.87	2.10	96.01	77.55	0.44	0.04	22.26	46.42	7.75	0.23	1.37
1.0	2.00	2.21	96.02	81.72	0.44	0.07	23.45	45.99	11.71	0.23	0.90
1.2	1.56	2.29	96.01	84.38	0.44	0.09	24.22	45.16	15.54	0.23	0.68
1.4	1.30	2.33	96.01	86.21	0.44	0.12	24.74	44.23	19.00	0.24	0.56
1.6	1.15	2.37	96.02	87.44	0.44	0.14	25.10	43.26	21.88	0.25	0.48

Table S4. Results of the simulation of the biogas two-stage upgrade process with the polyimide membrane (UBE, UMS-A5) and the pressure in the first stage of 0.8 MPa.

p_F	A	V_{BM}	$[CH_4]$	Recovery (CH_4)	P_{S1}	P_{S2}	P_{BM}	Power excess	Membrane productivity	Specific energy	Membrane specific area
MPa	m^2	m^3 (STP) h^{-1}	vol.%	%	kW_{el}	kW_{el}	kW_{th}	$kW_{th}kW_{el}^{-1}$	kW_{th} m^{-2}	kW_{el} m^{-3} (STP)	m^2 h m^{-3} (STP)
0.8	2.63	2.11	96.01	78.07	0.53	0	22.41	41.94	8.52	0.25	1.24
1.0	1.79	2.22	96.01	82.10	0.53	0.03	23.56	42.00	13.13	0.25	0.81
1.2	1.37	2.29	96.02	84.61	0.53	0.05	24.28	41.58	17.67	0.25	0.60
1.4	1.13	2.34	96.00	86.44	0.53	0.07	24.81	41.05	22.04	0.26	0.48
1.6	0.98	2.37	96.03	87.60	0.53	0.09	25.14	40.38	25.69	0.26	0.41

Table S5. Results of the simulation of the biogas one-stage upgrade process with the polysulfone membrane (Air Products, PRISM PA1020).

p_F	A	V_{BM}	[CH_4]	Recovery (CH_4)	P_{S1}	P_{S2}	P_{BM}	Power excess	Membrane productivity	Specific energy	Membrane specific area
MPa	m²	m³ (STP) h⁻¹	vol.%	%	kW_{el}	kW_{el}	kW_{th}	$kW_{th}kW_{el}^{-1}$	kW_{th} m^{-2}	kW_{el} m^{-3} (STP)	m^2 h m^{-3} (STP)
0.4	56.67	0.82	96.01	30.11	0.32	0	8.64	26.64	0.15	0.40	69.51
0.5	35.15	1.15	96.00	42.59	0.39	0	12.22	31.51	0.35	0.34	30.48
0.6	24.24	1.38	96.02	50.83	0.44	0	14.59	32.96	0.60	0.32	17.62
0.7	17.66	1.55	96.01	57.30	0.49	0	16.45	33.50	0.93	0.32	11.38
0.8	13.64	1.67	96.01	61.78	0.53	0	17.73	33.19	1.30	0.32	8.15
0.9	10.91	1.77	96.01	65.25	0.57	0	18.73	32.64	1.72	0.32	6.17
1.0	8.97	1.84	96.01	68.04	0.61	0	19.53	32.01	2.18	0.33	4.87
1.1	7.60	1.90	96.03	70.03	0.64	0	20.10	31.22	2.64	0.34	4.01
1.2	6.57	1.94	96.04	71.65	0.68	0	20.57	30.46	3.13	0.35	3.39

Table S6. Results of the simulation of the biogas two-stage upgrade process with the polysulfone membrane (Air Products, PRISM PA1020) and the pressure in the first stage of 0.4 MPa.

p_F	A	V_{BM}	[CH_4]	Recovery (CH_4)	P_{S1}	P_{S2}	P_{BM}	Power excess	Membrane productivity	Specific energy	Membrane specific area
MPa	m²	m³ (STP) h⁻¹	vol.%	%	kW_{el}	kW_{el}	kW_{th}	$kW_{th}kW_{el}^{-1}$	kW_{th} m^{-2}	kW_{el} m^{-3} (STP)	m^2 h m^{-3} (STP)
0.4	56.48	0.82	96.00	30.31	0.32	0	8.70	26.82	0.15	0.40	68.80
0.6	30.88	1.29	96.02	47.52	0.32	0.05	13.64	36.83	0.44	0.29	23.99
0.8	22.66	1.53	96.02	56.52	0.32	0.08	16.22	39.96	0.72	0.27	14.81
1.0	19.07	1.67	96.01	61.81	0.32	0.11	17.74	40.74	0.93	0.26	11.40
1.2	17.24	1.76	96.03	65.00	0.32	0.14	18.66	40.49	1.08	0.26	9.79

Table S7. Results of the simulation of the biogas two-stage upgrade process with the polysulfone membrane (Air Products, PRISM PA1020) and the pressure in the first stage of 0.6 MPa.

p_F	A	V_{BM}	[CH_4]	Recovery (CH_4)	P_{S1}	P_{S2}	P_{BM}	Power excess	Membrane productivity	Specific energy	Membrane specific area
MPa	m^2	m^3 (STP) h^{-1}	vol.%	%	kW_{el}	kW_{el}	kW_{th}	$kW_{th} kW_{el}^{-1}$	kW_{th} m^{-2}	kW_{el} m^{-3} (STP)	m^2 h m^{-3} (STP)
0.6	24.14	1.38	96.01	51.04	0.44	0	14.65	33.09	0.61	0.32	17.47
0.8	15.57	1.64	96.01	60.48	0.44	0.03	17.36	36.44	1.11	0.29	9.51
0.9	13.42	1.72	96.02	63.42	0.44	0.05	18.20	37.09	1.36	0.29	7.81
1.0	11.83	1.79	96.00	66.02	0.44	0.06	18.95	37.58	1.60	0.28	6.62
1.1	10.72	1.84	96.00	67.93	0.44	0.07	19.50	37.74	1.82	0.28	5.83
1.2	9.92	1.88	96.01	69.37	0.44	0.09	19.91	37.70	2.01	0.28	5.28

Table S8. Results of the simulation of the biogas two-stage upgrade process with the polysulfone membrane (Air Products, PRISM PA1020) and the pressure in the first stage of 0.8 MPa.

p_F	A	V_{BM}	[CH_4]	Recovery (CH_4)	P_{S1}	P_{S2}	P_{BM}	Power excess	Membrane productivity	Specific energy	Membrane specific area
MPa	m^2	m^3 (STP) h^{-1}	vol.%	%	kW_{el}	kW_{el}	kW_{th}	$kW_{th} kW_{el}^{-1}$	kW_{th} m^{-2}	kW_{el} m^{-3} (STP)	m^2 h m^{-3} (STP)
0.8	13.64	1.67	96.01	61.76	0.53	0	17.73	33.18	1.30	0.32	8.16
0.9	11.37	1.76	96.00	65.02	0.53	0.01	18.66	34.40	1.64	0.31	6.46
1.0	9.85	1.82	96.01	67.38	0.53	0.02	19.34	35.12	1.96	0.30	5.40
1.1	8.73	1.88	96.01	69.32	0.53	0.02	19.90	35.61	2.28	0.30	4.65
1.2	7.91	1.92	96.02	70.78	0.53	0.03	20.31	35.87	2.57	0.30	4.13
